# A Deletion Variant of Human Factor VIII Displaying Low Immunogenicity in a Murine Model of Hemophilia A

**DOI:** 10.3390/ijms262412093

**Published:** 2025-12-16

**Authors:** Erika de Simone Molina, Theri Leica Degaki, Mari Cleide Sogayar, Marcos Angelo Almeida Demasi

**Affiliations:** 1 Cell and Molecular Therapy NUCEL Group, School of Medicine, University of São Paulo, São Paulo 02146-903, SP, Brazil; 2 Biochemistry Department, Chemistry Institute, University of São Paulo, São Paulo 05508-000, SP, Brazil

**Keywords:** factor VIII, procoagulant factor, immunogenicity, bioengineering, recombinant

## Abstract

The therapeutic clotting factor VIII (FVIII) is known for its particular immunogenicity, with nearly 30% of hemophilic patients developing neutralizing antibodies against the infused protein. The root cause of this immunogenicity is still not well understood, but intrinsic factors, such as FVIII byproducts, have been linked to the immunological response elicited. Bioengineering of the FVIII molecule has been improving its recombinant (rhFVIII) production in many aspects, mainly enhancing its expression and stability. Assessment of immunogenicity for novel recombinant isoforms is crucial for further development and scaling-up processes, particularly due to the unpredictable antigenic properties and their impact on neutralizing antibody formation. In the present study, we describe a bioengineered human recombinant FVIII (rhFVIII-H6A), which induces lower immunogenicity in a murine model of hemophilia A. The rhFVIII-H6A product is characterized by a B-domain-deleted heavy chain (HCh), with the C-terminal of the B-domain fused to the light chain (BΔ-LCh). Compared to plasma-derived FVIII (pdFVIII) and rhFVIII reference products, treating hemophilic mice with rhFVIII-H6A induced lower levels of anti-FVIII antibody formation, including those with inhibitory neutralizing activity, while no difference was observed in the functional activity of rhFVIII-H6A in reverting the in vivo hemophilia phenotype. In addition, our results indicate that deleting the major part of the B-domain from the HCh might lower the immunogenicity of novel rhFVIII products.

## 1. Introduction

Factor VIII (FVIII) replacement therapy employs either FVIII concentrates from blood plasma or, alternatively, recombinant human FVIII, which is the standard of care for managing hemophilia A, a genetic bleeding disorder caused by plasmatic deficiency of this procoagulant factor. Recombinant human FVIII (rhFVIII), especially plasma/albumin-free third-generation rhFVIII products, represents a safer alternative to plasma-derived products due to a reduced risk of transmission of blood-borne pathogens. High costs are associated with rhFVIII production, since the expression yields are approximately two to three orders of magnitude lower than those achieved with recombinant proteins of similar sizes, such as the recombinant human Factor V [1]. In addition, FVIII is known for its particular immunogenicity, with 25–35% of hemophilic patients developing neutralizing alloantibodies against the infused FVIII [2,3]. Alloantibody formation directly interferes with the safety and efficacy of therapeutic products, affecting their toxicity, pharmacokinetic, and pharmacodynamic parameters. The risk of alloantibody development and the severity of the clinical effects vary among therapeutic proteins, examples including insulin, growth hormone, granulocyte-macrophage colony-stimulating factor (GM-CSF), erythropoietin, and interferons [4]. FVIII alloantibodies neutralize the FVIII functional activity by affecting the pharmacokinetic parameters, completely inhibiting drug activity. The alloantibodies to therapeutic human factor VIII in hemophilia A represent the major complication associated with FVIII replacement therapy [5,6]. Accordingly, therapeutic bioengineered molecules have been subjected to strict regulatory demands, among which are immunogenicity studies, and these molecules have been strategically assessed during their different product development stages. Genetically modified animal models have been useful for the evaluation of the immunogenic potential of several biopharmaceuticals in pre-clinical trials [7]. Hemophilia A mice, produced by targeted disruption of the *fviii* gene, have been used as a model system to study the immunogenicity of human FVIII [8]. After serial intravenous injections of human FVIII using a dose per body mass similar to that of humans, hemophilia A mice develop inhibitors [9]. The inhibitor response in hemophilia A mice includes antibodies that recognize the immune-dominant Arg484-Ile508 A2 epitope, which is recognized by human hemophilia A patients [10,11], although the extent to which other B cell epitopes in the murine model and human hemophilia are similar is still unknown. Overall, hemophilia A mice constitute a powerful model system to study rhFVIII immunogenicity and dissect the eventual immune responses. In the present study, we highlight the assessment of immunogenicity during the development of a B-domain-deleted (BDD) recombinant FVIII product produced in CHO-DG44 cells [12].

## 2. Results

### 2.1. Production of the Bioengineered Factor VIII (rhFVIII-H6A) Presenting an Artificial Light Chain (LCh) Fused to Part of the B-Domain

**Figure 1:**
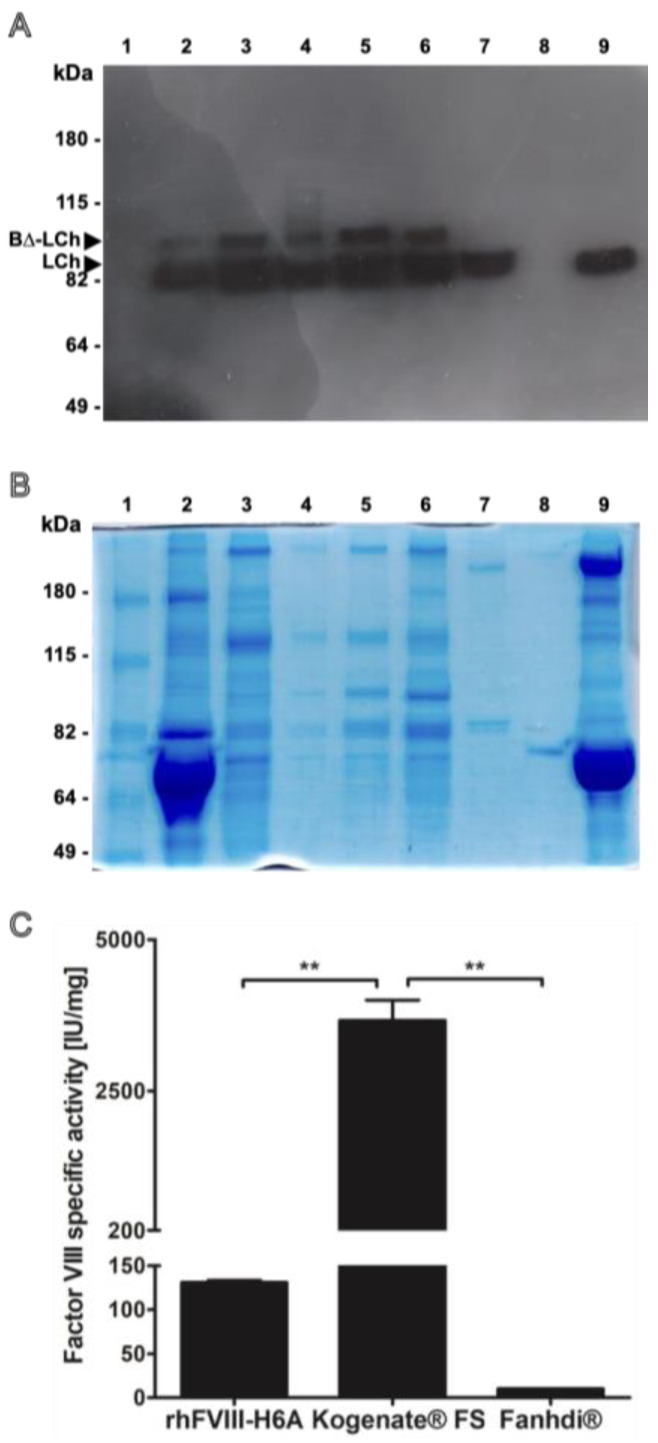
Purity, structural integrity analysis, and specific activity of the recombinant Factor VIII produced in our laboratory (rhFVIII-H6A) compared to reference commercial products. (**A**) Factor VIII light chain (LCh) Western blot analysis of the pool of samples derived from media conditioned by the H6A cell clone (well 2) (0.04 IU); after the anion exchange chromatography step (well 3) (0.07 IU); after the affinity chromatography step (well 4) (0.13 IU); retained fractions after the 100 kDa ultrafiltration step (well 5) (0.49 IU); rhFVIII-H6A final formulation (well 6) (0.5 IU); recombinant product Kogenate^®^ FS (well 7) (0.5 IU); and plasma derived Fanhdi^®^ (well 9) (0.5 IU). Water in well number 8. (**B**) Evaluation of the protein mixture complexity was carried out for the same samples described in (**A**) using 8% denaturing polyacrylamide gel electrophoresis, followed by staining with Coomassie blue G-250 colloidal using 2 IU of formulated rhH6A (well 6); a total of 2 IU of rhFVIII Kogenate^®^ FS (well 7) and 1 IU pdFVIII Fanhdi^®^ (well 9). Molecular weight ladder was applied in well number 1. (**C**) Factor VIII specific activity obtained for formulated rhFVIII-H6A. Specific activity was quantified based on the chromogenic in vitro Factor VIII activity assay and protein quantification using the colorimetric bicinchoninic acid assay. ** 0.01 < *p* < 0.05.

The recombinant human factor VIII (rhFVIII-H6A) was produced in our laboratory by employing the CHO-DG44-derived H6A cell clone, as previously described by Demasi, 2016 [13]. The H6A FVIII variant was described initially by Yonemura and colleagues [14] and contained a B-domain-deleted HCh and an artificial light chain (BΔ-LCh, described by Chen, 1999 [15]) fused to part of the B-domain. The rhFVIII-H6A product was purified from media conditioned for 24 to 48 h, and recovery was monitored throughout the purification procedure. Overexpression of the rhFVIII-H6A product is associated with the production of two main protein bands, namely one doublet of 80 kDa, corresponding to the fully proteolytically processed LCh isoforms starting at Glu1649 and Asp1658, and a 90 kDa protein band, corresponding to the proteolytically unprocessed B-LCh primary translation product starting at Asp1563 [11]. Both species are biologically active and expected in recombinant FVIII preparations. The doublet observed in rhFVIII-H6A is, therefore, consistent with known FVIII maturation heterogeneity and does not indicate degradation or impurity. In contrast, the LCh electrophoretic profile of pdFVIII (Fahndi^®^) and of the full-length rhFVIII (Kogenate^®^ FS) is characterized by the detection of only one 80 kDa protein band. The similar banding appearance between rhFVIII-H6A and pdFVIII is likely due to co-migrating protein species of comparable molecular weights rather than identical composition, since the pdFVIII preparation contains plasma proteins such as von Willebrand factor and albumin, while rhFVIII-H6A may include traces of host-cell proteins, proteins from fetal bovine serum used to supplement culture media, and formulation excipients. On the other hand, the electrophoretic profile of rhFVIII (Kogenate^®^ FS) is characterized by weak or absent bands due to its very high purity and specific activity (≈4500 IU/mg), which lead to minimal total protein content per IU and, hence, faint staining under Coomassie conditions. Finally, we specify that the faint signal in lane 8 corresponds to an extravasation of the large amount of albumin present in line 9. The rhFVIII-H6A-specific activity was assessed throughout the purification and formulation steps. The final formulated rhFVIII-H6A preparation was obtained after five batches and independent cycles of purification (Supplementary Table S1). The specific activity was compared to that of both reference products employed in this work, namely pdFVIII and rhFVIII. The purification protocol yielded an rhFVIII-H6A preparation displaying an intermediate specific activity value (135 IU/mg) when compared to pdFVIII (10 IU/mg) and rhFVIII (4500 IU/mg) (Figure 1).

### 2.2. Low Immunogenicity of rhFVIII-H6A in a Murine Model of Hemophilia A

**Figure 2:**
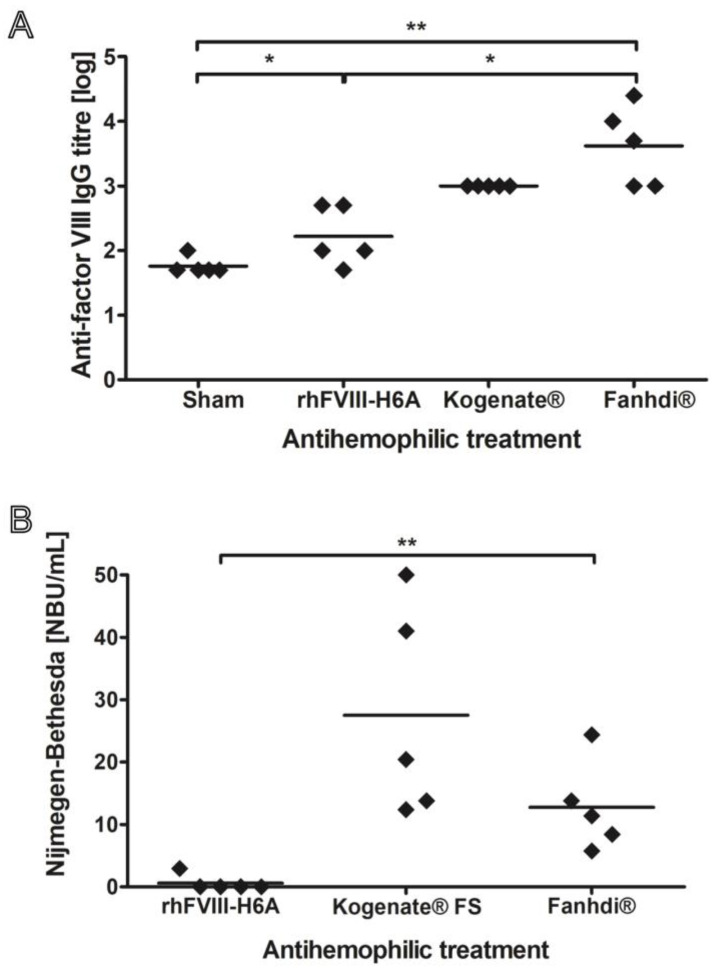
Alloantibody quantification in plasma of hemophilic mice after weekly treatments with the bioengineered Factor VIII (rhFVIII-H6A) compared to reference products. The animal model of severe hemophilia A was treated with four weekly administrations of vehicle, named Sham, or 50 IU/Kg of rhFVIII-H6A produced in our laboratory, commercial rhFVIII (Kogenate^®^ FS), or commercial pdFVIII (Fanhdi^®^). Five weeks after the beginning of the treatment, anti-factor VIII antibodies were quantified in the plasma samples (N = 5 per group). (**A**) Scatter plot of total anti-factor VIII antibodies, which were quantified by immunoenzymatic assay. (**B**) Scatter plot of anti-factor VIII neutralizing antibodies, which were quantified using the Nijmegen–Bethesda assay. One-way nonparametric analysis of variance followed by multiple comparison test (* 0.01 < *p* < 0.05, ** 0.001 < *p* < 0.01).

We first evaluated the immunogenicity of the rhFVIII-H6A isoform in comparison with highly purified full-length human rhFVIII (~4500 IU/mg). Since our rhFVIII-H6A preparation displayed a specific activity of approximately 130 IU/mg, indicating that only approximately 3% of the protein content is represented by FVIIII, we also included the pdFVIII commercial preparation, which has a specific activity of approximately 10 IU/mL, with only 0.2% of the protein content being represented by FVIII. In this sense, the design of our study was to compare the immunogenicity of our product with that of two reference products under equivalent pharmacodynamic conditions, thus producing a comparable hemostatic correction in FVIII-deficient mice and mimicking the clinical situation in which treatment decisions are based on functional activity (IU/kg) rather than protein mass. Although testing at equivalent specific activities was not included in this study, an investigation of the rhFVIII-H6A variant at doses normalized by protein mass content could provide complementary information about intrinsic antigenicity, independent of biological activity. The immunization protocol involved treatment by retro-orbital injection of six- to eight-week-old *fviii* knockout mice once a week with 50 IU/Kg of pdFVIII, rhFVIII, rhFVIII-H6A, or vehicle (N = 5 per group) for four weeks. The 50 IU/kg dose was elected because it corresponds to the dose regimen employed in the prophylactic treatment of hemophilic patients. Five weeks after beginning this treatment, plasma samples from the treated groups were evaluated for reactivity against rhFVIII (Figure 2). Total anti-rhFVIII antibody titers in the plasma samples were assessed ex vivo by ELISA. Figure 2A shows that the rhFVIII and pdFVIII preparations induced significantly higher values of anti-rhFVIII IgG titers (1000 ± 0 and 8400 ± 9990, respectively) than those observed in PBS-treated (Sham) mice (60 ± 22) (*p* < 0.05 for rhFVIII and *p* < 0.01 for pdFVIII). The titers of total anti-rhFVIII antibodies found in the plasma of mice treated with rhFVIII-H6A were numerically lower, though not statistically significant (250 ± 229), than those observed with rhFVIII and pdFVIII. Although the rhFVIII-H6A group presented markedly reduced total average anti-rhFVIII titers when compared to pdFVIII and rhFVIII (4 and 34 times lower, respectively), only pdFVIII induced a significantly higher anti-FVIII response (*p* < 0.05). Additionally, the average titer of the rhFVIII-H6A group was higher than that of the control group, but it was also not possible to establish statistical significance. These results suggest that the reduced immunogenicity observed for rhFVIII-H6A may be partly related to its structural features that limit the presence or exposure of epitopes associated with the inactive pro-FVIII heterodimer isoform. Figure 2B shows that all animals (N = 5 for each group tested) treated with either rhFVIII or pdFVIII presented a positive (NBU/mL > 0.5) Nijmegen–Bethesda titer of rhFVIII inhibitors, displaying a mean of 28 ± 17 NBU/mL and 13 ± 7 NBU/mL, respectively. Remarkably, only one animal (N = 5) treated with rhFVIII-H6A presented a positive Nijmegen–Bethesda titer (2.5 NBU/mL). The group of animals treated with rhFVIII-H6A displayed a mean of 0.5 ± 1.3 BU/mL, a value that is markedly (56 and 26 times) lower than that observed for rhFVIII and pdFVIII, although not statistically significant when compared to the latter group (Figure 2B). Together, these results indicate a trend for a markedly decreased anti-FVIII immune response elicited by the rhFVIII-H6A isoform, suggesting that this isoform constitutes a less immunogenic FVIII configuration.

### 2.3. In Vivo Functional Activity Evaluation of rhFVIII-H6A

**Figure 3:**
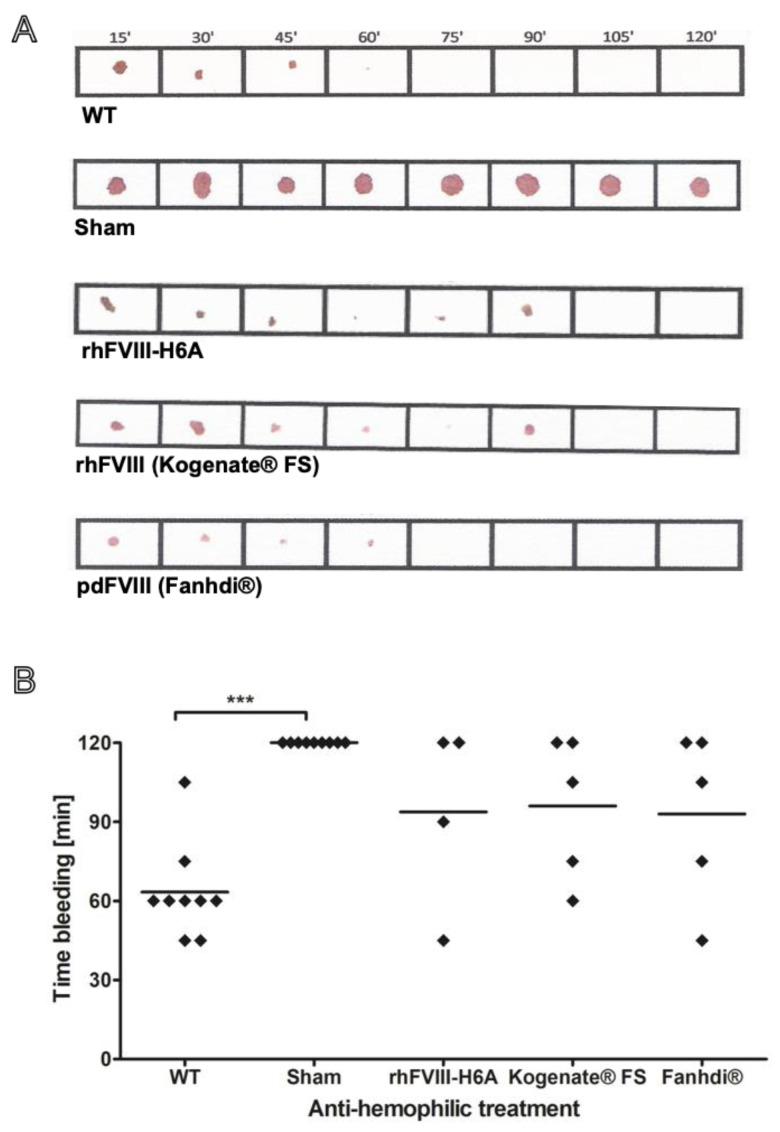
Functional in vivo activity of the bioengineered factor VIII produced in our laboratory (rhFVIII-H6A) compared to reference products. The anti-hemophilic effect was assessed by inducing a hemorrhagic event in Factor VIII knockout mice previously treated with factor VIII. The reversion of severe hemophilia A phenotype was estimated based on an assay involving tail bleeding interruption, indicating stable clot formation. The same background wild-type (WT) mice were used as a positive control of physiologic clot formation. WT animals were treated with vehicle (N = 9), and hemophilic animals were treated with vehicle, named Sham (N = 9), or 50 IU/Kg of rhFVIII-H6A (N = 4), commercial rhFVIII (Kogenate^®^ FS) (N = 5), or commercial pdFVIII (Fanhdi^®^) (N = 5). After 10 min of retro-orbital injections, a surgical blade was used to transect the animal’s tail at a level of 1 mm in diameter. The bleeding time was recorded on filter paper every 15 min until reaching 120 min, when the experiment was interrupted by tail cauterization of the Sham group. (**A**) Representative bleeding records obtained on paper filters for WT, rhH6A, and reference products. (**B**) Scatter plotting of bleeding time values. One-way analysis of variance followed by multiple comparison test (*** *p* < 0.001).

The in vivo hemostatic activity of FVIII was assessed using a murine model of severe hemophilia A through a tail bleeding hemostatic assay, based on blood clotting [14]. We evaluated the proportion of animals presenting a full hemostatic response to an intermediate dose (50 IU/kg) of FVIII within the 120 min of observation. Hemophilic mice injected with a 50 IU/kg dose of FVIII presented a partial hemostatic response, which is associated with approximately 60–70% probability to present a full hemostatic response within 120 min. Compared to the reference products pdFVIII (Fahndi^®^) and rhFVIII (Kogenate^®^ FS), the rhFVIII-H6A product presented a similar in vivo functional hemostatic activity (Figure 3).

## 3. Discussion

Inhibitory anti-FVIII antibody formation renders treatment of hemophilia A patients ineffective, representing the most severe complication, resulting in the need for new therapies with bypassing agents and cumbersome and expensive immune tolerance induction protocols [16,17]. Several factors related to the production and purification of therapeutic proteins impact their immunogenic potential, i.e., amino acid sequence and the structure of the molecule; post-translational modifications, such as glycosylation profile and proteolytic processing; the presence of impurities; the physical state of the molecules; and the method utilized for viral inactivation [17,18,19,20]. The root cause of FVIII immunogenicity is unclear, including potential contributions of some dissimilarities among rhFVIII products within their bioengineered chemical structure and post-translational modification patterns. In the present work, we investigated a bioengineered rhFVIII produced, purified, and formulated in our lab. To this rhFVIII, we observed a full hemostatic response in hemophilic mice treated with an intermediate dose of FVIII when they were challenged with an injury, demonstrating in vivo hemostatic activity similar to that of pdFVIII and rhFVIII reference products. Mainly, our objective was to conduct an initial, mechanistic evaluation of the immunogenic profile of the newly engineered rhFVIII-H6A molecule relative to two well-established benchmark products representing distinct reference categories, namely (1) a native full-length recombinant FVIII (Kogenate^®^ FS) and (2) a plasma-derived FVIII (Fanhdi^®^). This design allowed us to position the rhFVIII-H6A immunogenicity within the known spectrum of recombinant versus plasma-derived preparations and to isolate the potential effect of partial B-domain deletion relative to the full-length molecule. Here, we evaluated the immunogenicity of FVIII products with different characteristics, including structure (full-length vs. BDD heterodimer), presence of vWF (von Willebrand factor, contained in pdFVIII), and purity level. Most rhFVIII products explore the deletion or truncation of the B-domain, being produced using non-human cell lines. When the immunogenicity of rhFVIII-H6A was evaluated, an unexpectedly low antibody formation was observed, including a low level of antibodies with inhibitory activity The contribution of structural domains to the immunogenicity of the BDD rhFVIII is unclear, so the minimal inactive pro-FVIII heterodimer rhFVIII-H6A was bioengineered by deleting the B-domain from the HCh [14], with part of it fused to the light chain (BΔ-LCh) [15]. Most anti-FVIII inhibitory antibodies recognize A2, C1, and C2 FVIII domains [21,22,23,24,25], while anti-B-domain antibodies demonstrate low inhibitory activity against FVIII [26]. Compared to other moroctocog alfa products (Refacto AF^®^ and Xyntha^®^), our recombinant product of rhFVIII-H6A presents only five putative glycosylation sites compared to the 25 sites present in the canonical FVIII and is produced using the CHO cell line. It is not clear whether this differential glycosylation pattern could contribute to the differential immunogenicity potential of B-domain-deleted rFVIII. Still, it is one structural aspect that could be further investigated since extensive evidence of the involvement of N-glycans in proteolytic degradation, aggregation, denaturation, pharmacodynamics, pharmacokinetics, and, particularly, immunogenicity of therapeutic proteins is available [27]. Exploring two hemophilia A experimental models, Lai et al. reported that the N-linked glycosylated rhFVIII produced in BHK cells (Kogenate^®^ FS) was more immunogenic than that produced in CHO cells (Xyntha^®^) and that incomplete occupancy of N-linked sites led to the formation of IgM- and IgG-FVIII immune complexes [28]. Moreover, Arthur et al. reported that non-human glycans can regulate anti-FVIII antibody formation in mice, and that BHK-derived rhFVIII possesses higher levels of non-human glycans compared with CHO-derived rhFVIII [29]. The immunogenicity of artificially generated FVIII aggregates in animal models indicates that size, quantity, nature, and structure are essential in initiating unwanted immune responses toward native FVIII [30]. However, a comparative analysis of six commercially available rhFVIII products, including BDD or truncated, revealed no association between molecular structure and sub-visible particles or high-molecular-weight protein species [31,32]. Molecular aggregation studies of three rhFVIII products indicated more variable amounts of aggregates after reconstitution of ReFacto AF^®^ and Kogenate^®^ than of Advate^®^ [33]. Protein aggregates have been linked to increased immune responses to the monomeric form of the protein [34,35,36]. Regarding rhFVIII, it has been considered that aggregated forms do not enhance the immunogenicity but act as a distinct antigen [37].

Another potential factor associated with FVIII immunogenicity is that pre-formed complexes containing vWF increase the half-life and decrease the FVIII immunogenicity in animal studies [38]. One possible mechanism would be that the degradation of FVIII–vWF complexes, carried out by spleen macrophages, prevents the uptake of FVIII by antigen-presenting cells [39,40,41,42]. In accordance, vWF blocks FVIII binding to the macrophage mannose receptor (CD206) in a dose-dependent manner [43], and an in vitro activation of FVIII-specific CD4^+^ T cells by dendritic cells is abrogated upon removal of the N-linked glycosylation site [44]. However, the notion that FVIII products containing VWF may be less immunogenic is controversial. Clinical evidence of inhibitors in hemophilia A indicates pdFVIII products with intact VWF may be less immunogenic [45], resulting in a lower incidence of inhibitors than rhFVIII [46,47]. Our data (Figure 2B) indicate a tendency for lower immunogenicity of pdFVIII compared to rFVIII, suggesting a potential effect of vWF in suppressing the immunogenic response toward FVIII. The physiological context associated with activation of the blood coagulation cascade leading to thrombin production and creating a pro-inflammatory context per se could also act as a trigger, generating signals that could stimulate an immune response to FVIII [48,49,50,51]. It has been postulated that the immunogenicity elicited by FVIII could be related, at least in part, to its function in potentiating the blood coagulation intrinsic pathway during the hemostatic response, thereby generating the inflammatory response context required to induce an immunological response to FVIII. According to this hypothesis, a combination of FVIII presence, which in hemophilic patients represents a protein for which tolerance may not have been completely induced, and a co-stimulation signal triggered by thrombin’s action would induce the formation of inhibitory antibodies to FVIII. Skupsky and colleagues [52] (2009) studied the immunogenicity of human heat-denatured FVIII in hemophilic mice and found that the denatured form was less immunogenic than the native form. In addition, these authors observed a decrease in lymphocyte response and in antibody formation against FVIII in hemophilic mice treated with the anticoagulant warfarin or the Hirudin 4 thrombin inhibitor. These data could suggest a role for thrombin formation through the procoagulant activity of FVIII, inducing co-stimulation of FVIII inhibitor formation. Interestingly, complexed or not to vWF, rhFVIII was shown not to present co-stimulatory signals for human dendritic cells [53].

However, Meeks and colleagues (2012) [54] suggested that the primary determinant of immunogenicity to factor VIII in a murine model is independent of its procoagulant function. In agreement, Gangadharan et al. reported that thrombin generation or associated coagulation processes do not modulate the anti-FVIII antibody response. Instead, these authors reported that warfarin anticoagulant treatment failed to reduce immunogenicity [55]. To enhance the study of anti-FVIII specific responses, FVIII was recently engineered by incorporating the model antigen ovalbumin to study CD4 T cell responses [56]. We investigated the in vivo hemostatic activity of the purified and formulated rhFVIII-H6A compared to the pdFVIII and rhFVIII reference products, observing similar hemostatic activity among these different sources of FVIII. At this point, we could not determine which factors could contribute to rhFVIII-H6A displaying unexpectedly low immunogenicity in a murine model of hemophilia A. The fact that the rhFVIII-H6A preparations were partially purified could be a confounding factor. However, it should also be mentioned that pdFVIII products contain many other proteins as well, which may influence their FVIII immunogenicity. Importantly and with some relevance to the context of decreasing the immunogenicity of rFVIII for better treatment of hemophilia A patients, we demonstrated that the treatment with the bioengineered rhFVIII-H6A (compared to plasma-derived and recombinant reference products) corrected the hemophilic phenotype in vivo, inducing very low levels of antibody formation and especially low FVIII neutralizing activity. This work would contribute to addressing the important unmet needs of current prophylaxis for hemophilia A, particularly in the research of factor FVIII products with lower immunogenicity. Overcoming the limitations resulting from the immunogenicity, the development of better and safer therapy products for the treatment of hemophilia A patients could be a new alternative.

A limitation of the present study is the absence of a direct comparison between rhFVIII-H6A and licensed B-domain-deleted recombinant FVIII (BDD-rFVIII) products, such as moroctocog alfa or turoctocog alfa. As acknowledged in our response to the reviewers, such head-to-head analyses would enhance the translational relevance of our findings by situating rhFVIII-H6A within the spectrum of clinically used BDD products. Direct inclusion of these commercial products was not feasible due to restricted research access and proprietary constraints as well as differences in manufacturing platforms that limit controlled experimental comparison. Future work should incorporate licensed BDD-rFVIII molecules to more precisely assess how rhFVIII-H6A compares to structurally related therapeutic FVIII products in terms of immunogenicity.

Published evidence indicates that B-domain deletion itself does not appear to be a major driver of immunogenicity. Several clinical studies and systematic reviews have shown that BDD and full-length rFVIII products present broadly comparable risks of inhibitor formation: Early prospective clinical studies in previously treated patients (PTPs) demonstrated no increased immunogenicity of BDD-rFVIII compared with full-length products [57] (Court et al., 2001). Post-marketing cohort analyses evaluating large product switches also reported no significant change in inhibitor incidence when patients transitioned from full-length to BDD-rFVIII [58] (Hay et al., 2015). Systematic reviews, including the work of Hassan et al. (2018) [59], concluded that, in PTPs, BDD-rFVIII is not associated with higher inhibitor risk relative to full-length products. Meta-analyses and reviews by Aledort (2011) [60] and Xi (2013) [61] emphasized that differences in immunogenicity among rFVIII products are more likely related to manufacturing processes, cell line origin, and glycosylation patterns than to the B-domain deletion. Across multiple studies summarized by Gouw et al. (2013) [62], BDD structure alone was not identified as a determinant of inhibitor development; instead, immunogenicity appeared to reflect product-specific biochemical features. Furthermore, mechanistic studies indicate that domains most commonly targeted by inhibitory antibodies—A2, C1, and C2—are preserved in both full-length and BDD rFVIII. Consistent with this, Samelson-Jones (2019) [63] discussed how protein-engineered FVIII molecules differ substantially in immunogenicity based on folding, domain stability, and glycan occupancy rather than the presence or absence of the B-domain.

Recent immunogenetic findings highlight that host factors may contribute more substantially to inhibitor risk than FVIII structure alone. Pratt et al. (2023) [64] demonstrated that F8 gene haplotypes may influence inhibitor development independent of the therapeutic FVIII product used. These data reinforce that the relationship between FVIII structure and inhibitor formation is multifactorial and cannot be attributed solely to the B-domain.

Collectively, the available literature supports the view that differences between full-length and BDD-rFVIII products arise predominantly from manufacturing platform, glycosylation profile, aggregation propensity, and post-translational modifications rather than from the structural absence of the B-domain itself. These findings emphasize the importance of investigating additional structural characteristics of rhFVIII-H6A—such as glycan occupancy, aggregation state, and the relative abundance of inactive pro-FVIII heterodimer—in future comparisons involving licensed BDD-rFVIII products.

## 4. Materials and Methods

### 4.1. Culture of CHO-DG44 Cell Clones Overexpressing rhFVIII

CHO-DG44 cells [12] were co-transfected with pIQID-HC and pIQID-LC *dhfr*-bicistronic expression plasmids [13] using the Lipofectamine 2000 reagent (Invitrogen Corporation, Carlsbad, CA, USA) for the selection of a cell clone named CHO-DG44-H6A, as described by Demasi, 2016 [13]: the amplified HC cDNA encodes the FVIII-contained 19-aa signal peptide and A1 and A2 domains (Ala1 to Arg740) [14]; the cDNA sequence encoding the derivative of FVIII LC (B-LC), described by Yonemura et al. [15], encodes the 19-aa FVIII signal peptide, the B-domain C-terminal portion (Asp1563 to Arg1648), and the entire LC (domains A3, C1, and C2) (Glu1649 to Tyr2332). The representative structure of FVIII is shown in the Visual abstract.

The human recombinant FVIII-producing CHO-DG44-H6A cells were expanded in HAM’s F12 medium (Invitrogen Corporation, Carlsbad, CA, USA) supplemented with 10% heat-inactivated fetal bovine serum (FBS) (Invitrogen Corporation, Carlsbad, CA, USA) supplemented with 100 µg/mL ampicillin, 100 µg/mL streptomycin, and 1.2 g/L sodium bicarbonate in a humidified atmosphere of 2% CO_2_ in air at 37 °C.

### 4.2. Purification of rhFVIII-H6A

Purification of *rhFVIII-H6A* was carried out from medium conditioned by the H6A clone for 24–48 h upon cell culturing as a monolayer using α-MEM supplemented with 7% FBS in three steps, namely two chromatographic steps and one ultrafiltration. The chromatographic steps were performed using the Akta Purifier UPC 100 system, controlled by the Unicorn 5.3 software (GE Healthcare, Uppsala, Sweden). Anion-exchange chromatography was carried out using prepacked 5 mL *Hitrap Q-Sepharose Fast Flow* columns (GE Healthcare, Uppsala, Sweden). Column equilibration was achieved with A1 buffer (5 mM CaCl_2_), and unbound proteins were washed out using 3.8% of the A2 buffer (5 mM CaCl_2_; 2 M NaCl) in the A1 buffer. Stepwise elution was carried out using three different NaCl concentrations, namely 5 CV of 26%, 5 CV of 50%, and 5 CV of 100% from the A2 buffer. Pseudo-affinity chromatography using prepacked 1 mL columns *Hitrap Heparin HP* (GE Healthcare) was employed. Equilibration was carried out with B1 buffer (150 mM NaCl; 5 mM CaCl_2_), and washing of unbound proteins was carried out sequentially with 1% and 24% of the B2 buffer (150 mM NaCl; 500 mM CaCl_2_) in the B1 buffer. The system was rewashed with 18 CV of 24% B2 buffer. Next, elution was performed using 100% B2 buffer. Samples were then 8-fold concentrated twice through ultrafiltration (1500× *g* at 4 °C) using Amicon Ultra-4 100 kDa (Millipore Corporation, Carrigtwohill, Ireland) with one step of dilution (1:7) in formulation solution (1.3% Sucrose; 25 mg/mL Glycine; 23 mM Histidine; 36 mEq/L NaCl; 80 μg/mL Polysorbate 80; 20 mM Imidazole; 0.06 µg/mL CuCl_2_; pH 7.3). Samples were sterilized using 0.22 µm membranes and snap-frozen. Representative chromatograms and typical recovery results are provided in the Supplementary Figures S1 and S2.

### 4.3. Quantification of Total Proteins

Total protein was quantified using the colorimetric assay based on bicinchoninic acid, namely the BCA Protein Assay commercial kit (Lot CI48587) from Pierce (ThermoFisher Scientific, Rockford, IL, USA), following the manufacturer’s instructions.

### 4.4. FVIII Chromogenic Assay

FVIII activity was quantified employing a chromogenic activity assay (Coatest SP4 FVIII, Chromogenix, Milan, Italy) using the acetic acid-stopped endpoint method according to the manufacturer’s instructions. All samples were diluted to yield values within the linear range of the assay.

### 4.5. Western Blot Analysis

Samples collected from all purification steps were fractionated with 8% SDS-polyacrylamide gel electrophoresis. The resolved proteins were blotted onto nitrocellulose membranes (Bio-Rad, Hercules, CA, USA), which were blocked overnight at 4 °C with 5% nonfat milk in TBS-T buffer containing 0.05% Tween 20. The blots were probed in TBS-T/milk with the anti-LCh (Chemicon Mab-038, Merck-Millipore, Burlington, MA, USA) (dilution 1:750) and then developed using (HRP)-conjugated secondary antibodies (Invitrogen) and ECL Plus chemiluminescent detection reagent (GE HealthCare, Piscataway, NJ, USA), according to the manufacturer’s instructions.

### 4.6. Reference Products

The plasma-derived factor VIII Fanhdi^®^ (Lot IBVC0XEXG1, Grifols Institute, Parets del Valles, Spain) was donated by the Brazilian Program of Coagulopathies from The Ministry of Health. The recombinant factor VIII Kogenate^®^ FS (Lot 27N2JP2, Bayer AG, Leverkusen, Germany) was donated by the Secretary of Health from the Federal District of Brazil.

### 4.7. Animals

FVIII knockout mice (B6;129S4-F8tm1Kaz/J strain), generated through targeted exon-6 disruption [8], were purchased from The Jackson Laboratory (Bar Harbor, ME, USA). FVIII wild-type mice (129J/Sv strain) were purchased from the Animal Facility of the University of São Paulo Biomedical Sciences Institute (ICB/USP, São Paulo, Brazil). All mice procedures were approved by the Ethics Committee on Animal Care and Use of the Chemistry Institute of the University of São Paulo (Protocol number 20/2012), being in accordance with the Brazilian National Council for Control of Animal Experimentation (CONCEA).

### 4.8. Functional Activity of FVIII

A quantitative tail bleeding hemostatic assay was carried out as previously described [65]. Hemophilic mice were anesthetized and treated with FVIII by retro-orbital injection. Ten minutes after FVIII administration, the tip of the mice’s tails was transected at 1 mm diameter using a ring template, and the time for coagulation was quantified. Bleeding was recorded every 15 min by blotting the blood accumulated on the mouse tail tip during the 15 min observational interval onto a 3 mm *Whatmann* cellulose filter paper.

### 4.9. Induction of Immunological Response to Factor VIII

Induction of immunological response was assessed using the murine model of severe hemophilia A. Mice were divided into four groups of five animals each (N = 5) for treatment with vehicle (formulation solution) or 50 IU/kg of *rhFVIII-H6A* or reference products rhFVIII (Kogenate^®^ FS) and pdFVIII (Fanhdi^®^). Mice received four weekly administrations by retro-orbital injections into the venous sinus using a 27-gauge needle, and five weeks from the beginning of the treatment, 180 µL of blood was collected directly into tubes containing 20 µL of 130 mM sodium citrate by puncturing the submandibular vein using an 18 G needle. Samples were centrifuged for 5 min at 2000× *g*, and plasma samples were immediately frozen for further analysis.

### 4.10. Dosage of Total Anti-Factor VIII Antibodies

Titers of anti-FVIII immunoglobulins G (IgG) were measured in duplicate for the plasma of hemophilic mice treated with *rhFVIII-H6A*, rhFVIII (Kogenate^®^ FS), pdFVIII (Fanhdi^®^), or vehicle by enzymatic immunoassay (ELISA). Ant-FVIII titers were assessed in 96-well Nunc MaxiSorp coated with rhFVIII Kogenate^®^ FS. Briefly, the coating was carried out with rhFVIII in 50 µL of carbonate buffer pH 9.4 for 16 h at 4 °C, followed by washing with 0.1% Tween 20 in phosphate-buffered saline solution (PBSA). Non-specific binding sites were blocked with 200 µL of 2% (*w*/*v*) bovine serum albumin (BSA) in PBSA at room temperature and a humid atmosphere for 2 h. The wells were washed three times using 0.1% Tween 20 and incubated for 2 h at 37 °C with 100 µL samples of plasma from hemophilic mice, which were serially diluted in blocking solution. Analysis was carried out with plasma dilution curves from animals treated with rhFVIII-H6A 1:25, 1:50, 1:100, 1:500, and 1:1000; Kogenate^®^ FS 1:100, 1:500, 1:1000, 1:5000, 1:10,000, and 1:25,000; Fanhdi^®^ 1:1000, 1:5000, 1:10,000, 1:25,000, 1:50,000, and 1:75,000; and vehicle 1:25, 1:50, 1:100, 1:500, and 1:1000. The wells were washed three times using 0.1% Tween 20 and then incubated with anti-mouse secondary antibody IgG from Invitrogen (Life Technologies, Burlingame, CA, USA) conjugated to peroxidase and diluted 1:2000 in blocking solution at room temperature and a humid atmosphere for 1 h and 30 min. The wells were then washed three times with 0.1% Tween 20, and reactions were revealed using the TMB ELISA kit solution (Thermo Fisher Scientific, Rockford, IL, USA), according to the manufacturer’s instructions. Absorbance was measured at 405 nm using a Molecular Devices SpectraMax M2 (LLC Molecular Devices, Sunnyvale, CA, USA). Antibody titers were estimated based on the highest dilution presenting a positive signal, defined as the absorbance value greater than 0.05, representing the mean of the negative control plus the standard deviation value multiplied by three.

### 4.11. Dosage of Total Inhibitory Factor VIII Antibodies

Titers of inhibitors were detected in the plasma of hemophilic mice treated with vehicle rhFVIII-H6A, rhFVIII (Kogenate^®^ FS), or pdFVIII (Fanhdi^®^) based on the Nijmegen-modified Bethesda assay [66]. Plasma samples from the animals previously treated with rhFVIII-H6A, Kogenate^®^ FS, Fanhdi^®^, or vehicle were first incubated at 58 °C for 90 min to inactivate any residual FVIII activity, followed by centrifugation at 4000× *g* for 2 min to remove any debris. Plasma samples were serially diluted in commercial human FVIII-deficient plasma (Affinity Biologicals Inc, Ancaster, ON, Canada). For the group of animals treated with rhH6A, samples were diluted 1:5 and 1:25; for Kogenate^®^ FS, samples were diluted 1:10 to 1:50; for Fanhdi^®^, samples were diluted 1:20, 1:30, 1:100, and 1:150; and for the vehicle, which was defined as ‘plasma-test’, samples were diluted 1:4 and 1:20. Triplicate samples containing only FVIII-deficient plasma were used as ‘plasma-control’ samples. ‘Plasma-test’ and ‘plasma-control’ samples were incubated for 2 h at 37 °C with an equal volume (45 µL) of 2 IU/mL of FVIII (Fanhdi^®^) in FVIII-deficient plasma buffered with 100 mM Imidazole at pH 7.4. After the incubation period, the FVIII activity was evaluated in ‘plasma-test’ and ‘plasma-control’ samples by the in vitro chromogenic assay. The FVIII activity obtained in the ‘plasma-test’ samples was expressed relative to that obtained in the ‘control plasma’ samples and then referred to as ‘remaining FVIII activity (%). Quantification of inhibitors was performed using linear regression on a semi-logarithmic scale, assuming that 100% of the remaining FVIII activity corresponded to 0.0 NBU/mL and that 50% of the remaining activity corresponded to 1.0 NBU/mL. The remaining activity values were only estimated between 25% and 75% from the FVIII activity of control plasma samples, since only this interval would linearly correlate to the presence of FVIII inhibitors.

### 4.12. Statistical Analysis

The different levels of total and inhibitory anti-factor VIII antibody formation among the plasma of hemophilic animals treated with rhFVIII-H6A, rhFVIII (Kogenate^®^ FS), pdFVIII (Fanhdi^®^), or vehicle (Sham) were compared through the nonparametric one-way analysis of variance Kruskal–Wallis test, followed by Dunn’s multiple comparison test using GraphPad 5.0 (GraphPad Software, San Diego, CA, USA). Statistical significance was set at *p* < 0.05. Data are expressed as the mean ± SD representing independent animals.

## Data Availability

The original contributions presented in this study are included in the article/Supplementary Materials. Further inquiries can be directed to the corresponding author.

## Supplementary Materials

The following supporting information can be downloaded at: https://www.mdpi.com/article/10.3390/ijms262412093/s1.
