# Supplementary material for: A Deletion Variant of Human Factor VIII Displaying Low Immunogenicity in a Murine Model of Hemophilia A
Source: Int J Mol Sci. 2025 Dec 16;26(24):12093. doi: 10.3390/ijms262412093 (PMC12733223; doi:10.3390/ijms262412093)
Supplement: Supplementary file 1 [file ijms-26-12093-s001.zip › ijms-3934703-supplementary.pdf]

Supplemental Materials

Supplementary Table S1: Purification of the recombinant factor VIII (rhH6A) produced in our laboratory. Activity levels (IU) were quantified based on the chromogenic *in vitro* factor VIII assay and total protein levels (mg) were quantified based on the colorimetric bicinchoninic acid assay. The specific activity was assessed in samples collected from the conditioned media, after the anion exchange chromatography, after the pseudo-affinity chromatography, after ultrafiltration, and from the final formulation. The final product was obtained from the mixture of five production and purification batches and 6x volume concentration. The yield was determined based on the ratio of factor VIII activity at each step and the conditioned media, multiplied by 100. The purification factor was determined based on the specific activity ratio at each step and the starting media. Protein levels after the ultrafiltration step were not determined.

Purification step	Activity (UI)	Protein (mg)	Specific activity (UI/mg)	Yield (%)	Purification factor
Conditioned medium	111.9	753.0	0.2	100	1
Ion exchange chromatography	37.0	10.1	3.7	33	25
Heparin affinity chromatography	25.6	0.2	150.9	23	1,016
100 kDa Ultrafiltration	8.6	*	*	8	*
Formulated rhFVIII-H6A product	54.9	0,42	131.7		

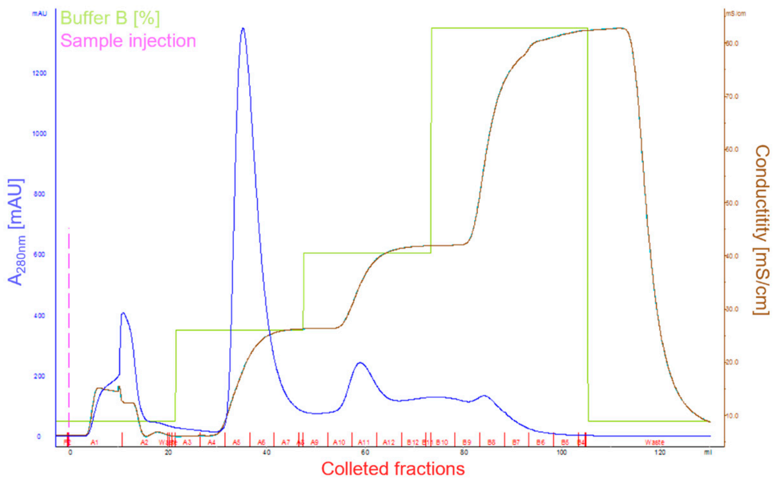
Buffer B [%]
Sample injection
A280nm [mAU]
Conductivity [mS/cm]
Colleted fractions

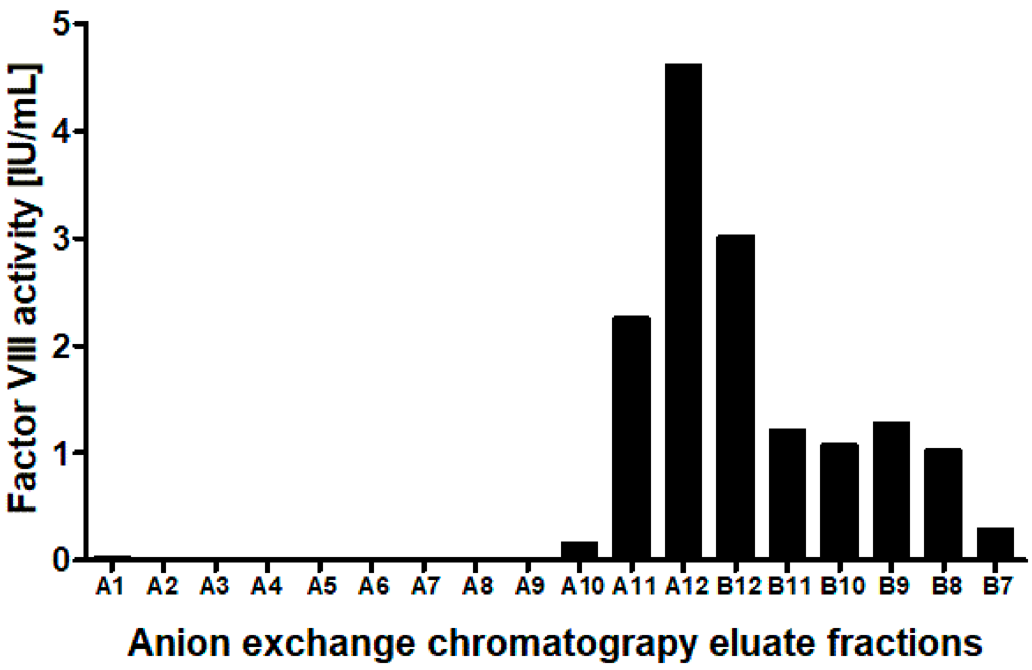
Factor VIII activity [IU/mL]
0
1
2
3
4
5
A1 A2 A3 A4 A5 A6 A7 A8 A9 A10 A11 A12 B12 B11 B10 B9 B8 B7
Anion exchange chromatograpy eluate fractions

Supplementary Figure S1. Purification of recombinant Factor VIII produced in our laboratory (rhFVIII-H6A) through anion exchange chromatography. (A) Typical chromatogram obtained by fractionating the culture medium (α-MEM without nucleosides supplemented with 7% FCS) conditioned by the H6A cell clone overproducing the Factor VIII by anion exchange chromatography associated with an isocratic elution regimen. The spectrophotometric absorption at 280nm (blue curve) indicates protein elution peaks. The brown curve indicates conductivity values associated with changes in salt concentration (green curve) during the elution step. The abscissa axis represents elution fractions (A1 to B4), each one corresponding to a 5 mL column volume. The left (Y1) ordinate axis represents the absorbance at 280 nm in milliunits (mAU) and on the right (Y2) is the conductivity (mS/cm). The vertical pink dashed line indicates the moment of sample injection in a 10 mL loop, with the remainder of the sample (~80 mL) injected using a peristaltic pump. The protein peak in fractions A1 and A2 represents the elution of the last 5 mL of proteins not bound to the column. **(B)** FVIII activity (IU/mL) was quantified by the chromogenic *in vitro* activity assay for all eluted fractions obtained during the procedure, being column permeates (A1-A2), washings (A3-A4) and elution samples (A5-B7). The elution fractions A11, A12 and B12, enriched in FVIII activity, were eluted with conductivity values between 30.7 and 41.9 mS/cm (brown curve).

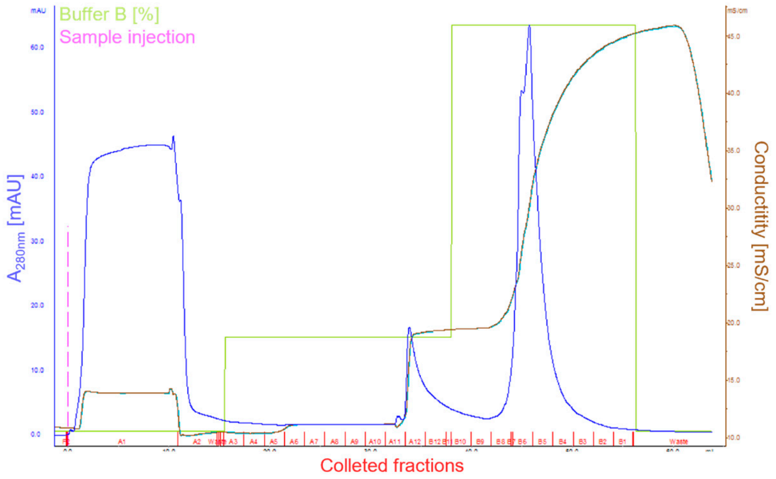

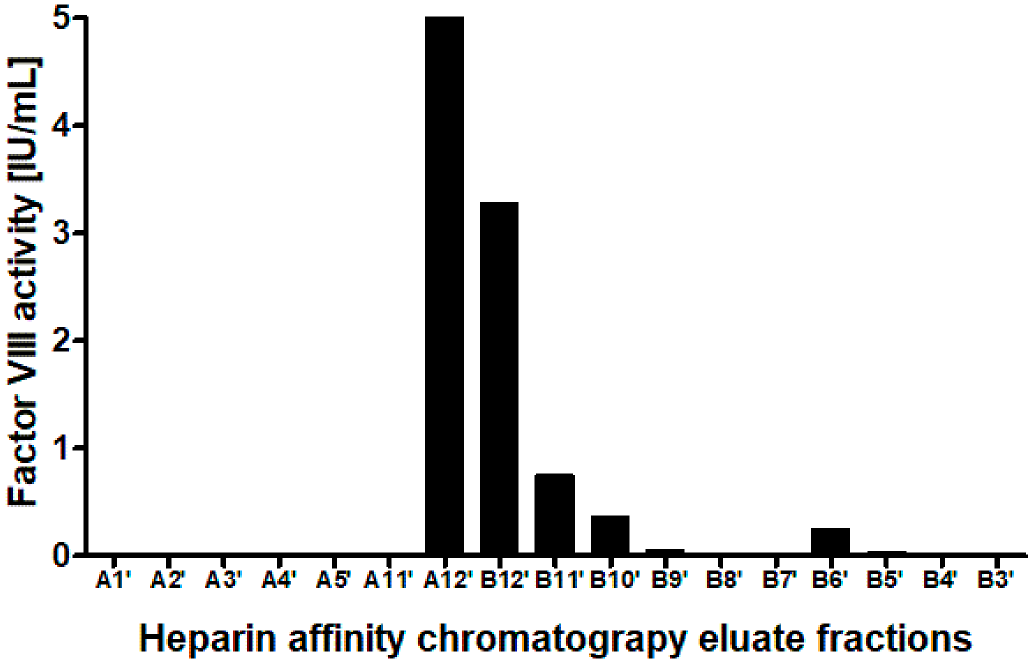

Supplementary Figure S2. Purification of recombinant factor VIII produced in our laboratory (rhFVIII-H6A) through heparin affinity chromatography. **(A)** Typical chromatogram obtained by heparin affinity chromatography of the fractions enriched in factor VIII activity after the anion exchange chromatography. The spectrophotometric absorption at 280 nm (blue curve) indicates protein elution peaks. The brown curve indicates conductivity values associated with changes in salt concentration (green curve) during the elution step. Elution fractions (A1' to A5' and A11' to B1'), each corresponding to a 1 mL column volume, are represented in the abscissa axis. The left (Y1) ordinate axis represents the absorbance at 280 nm in milliunits (mAU) and on the right (Y2) is the conductivity (mS/cm). The vertical pink dashed line indicates the moment of sample injection in a 10 mL loop, with the remainder of the sample (~35 mL) being injected using a peristaltic pump. The protein peak in fractions A1 and A2 represents the elution of the last 1 mL of proteins, which were not bound to the column. **(B)** FVIII activity (IU/mL) was quantified using the chromogenic assay of *in vitro* activity for all eluted fractions obtained during the procedure, namely, column permeate (A1'), washings (A2'-A5) and elution samples (A11'-B3'). The elution fractions A12' and B12', enriched in FVIII activity, were eluted with conductivity values between 11.9 and 19.4 mS/cm (brown curve).
